# Tuberculous tenosynovitis as the initial presentation of disseminated tuberculosis in a patient with uncontrolled diabetes: A case report and review of literature

**DOI:** 10.1016/j.idcr.2026.e02525

**Published:** 2026-02-20

**Authors:** Eunice Susan Thomson, Merlin Moni, Balu Chandrababu, Anil Kumar, Dipu T. Satyapalan, Nivedita Suresh, Anna Kurian, Kiran G. Kulirankal

**Affiliations:** a Department of General Medicine, Jubilee Mission Medical College and Research Institute, Thrissur, Kerala, India; b Division of Infectious Diseases, Department of General Medicine, Amrita Institute of Medical Sciences, Kochi, Kerala, India; c Department of Orthopaedics, Amrita Institute of Medical Sciences, Kochi, Kerala, India; d Department of Microbiology, Amrita Institute of Medical Sciences, Kochi, Kerala, India; e Department of Pathology, Amrita Institute of Medical Sciences, Kochi, Kerala, India

**Keywords:** Tuberculous tenosynovitis, Rice bodies, Disseminated tuberculosis, Extrapulmonary tuberculosis, Diabetes mellitus

## Abstract

A middle-aged woman with uncontrolled type 2 diabetes mellitus presented with pain in the right arm for 1 year, multiple gradually increasing painful swellings across her right wrist for 6 months, and intermittent fever of 3 months duration. She developed painful flexion and extension of the right ring and middle fingers as well as the right wrist. Physical examination revealed flexion contractures in the right and middle finger, mobile and non-tender axillary lymph nodes. Blood examination revealed an elevated erythrocyte sedimentation rate. An externally performed magnetic resonance imaging of the right wrist revealed multiple inflammatory swellings involving several tendon sheaths. A tendon sheath biopsy revealed multiple rice bodies within the tendon sheath. The tissue sample tested positive for acid-fast bacilli (AFB), and nucleic acid amplification test (NAAT/ Gene Xpert) result was positive. Chest radiography revealed bilateral infiltrates. The patient was initiated on anti-tubercular therapy with which she improved symptomatically.

## Highlights

• Rare presentation of extrapulmonary tuberculosis presenting as wrist tenosynovitis.
• Uncontrolled diabetes predisposed to disseminated tuberculosis.
• Definitive diagnosis often requires histopathological examination
• Gene Xpert confirmed *M. tuberculosis* in both tendon and sputum samples
• Early anti-tubercular therapy led to full functional recovery

## Introduction

Tuberculosis (TB), a chronic infectious disease caused by Mycobacterium tuberculosis, is one of the most important public health concerns in India and other endemic regions [1]. Although pulmonary involvement is the most common manifestation, extrapulmonary tuberculosis (EPTB) accounts for approximately 15–20 % of all TB cases, particularly among immunocompromised individuals [2]. Musculoskeletal TB is an uncommon form of EPTB, representing about 1–3 % of cases, and involvement of tendon sheath, refers to as tuberculous tenosynovitis, is especially rare [3].

Tuberculous tenosynovitis most frequently involves the hand and wrist and typically follows an indolent course with minimal systemic symptoms [4]. Owing to its nonspecific clinical presentation and radiological resemblance to inflammatory or degenerative conditions, diagnosis is often delayed. Microbiological confirmation may be challenging, and definitive diagnosis frequently relies on histopathological examination [3], [4]. As a result, patients may present late, sometimes with extensive local disease or dissemination.

Diabetes mellitus is a well-recognised risk factor for both pulmonary and extra pulmonary TB and is associated with an increase likelihood of atypical and disseminated presentations [5].Impaired cell mediated immunity, altered macrophage function, and dysregulated cytokine response in diabetic individuals contribute to increased susceptibility and delayed diagnostics [2]. Other risk factors include immunosuppression due to medications and human immunodeficiency virus (HIV) infection [2]. Diagnosis requires a combination of imaging studies and microbiological or histopathological identification of *Mycobacterium tuberculosis* from tissue samples [3].

Treatment requires prolonged therapy with anti-TB medications for a duration of 6–12 months [3].Healthcare workers should be aware of atypical presentations, especially in endemic regions, as early recognition and intervention are crucial for preventing long-term impairment.

## Case report

A middle aged female, with a history of type 2 diabetes mellitus (HbA1c-10) was referred to our hospital with complaints of pain across her right arm for 1-year, recurring multiple painful swelling of her dorsal surface of right wrist for 6 months, and intermittent low grade fever with chills of 3 months duration. She was unable to flex or extend her middle and ring fingers and had only a minimal range of motion of the right wrist, which impaired her day-to-day activities.

The patient gave no history of trauma, prior history of TB, weight loss, cough, night sweats, exposure to TB patients, or any respiratory symptoms.

**Fig. 1:**
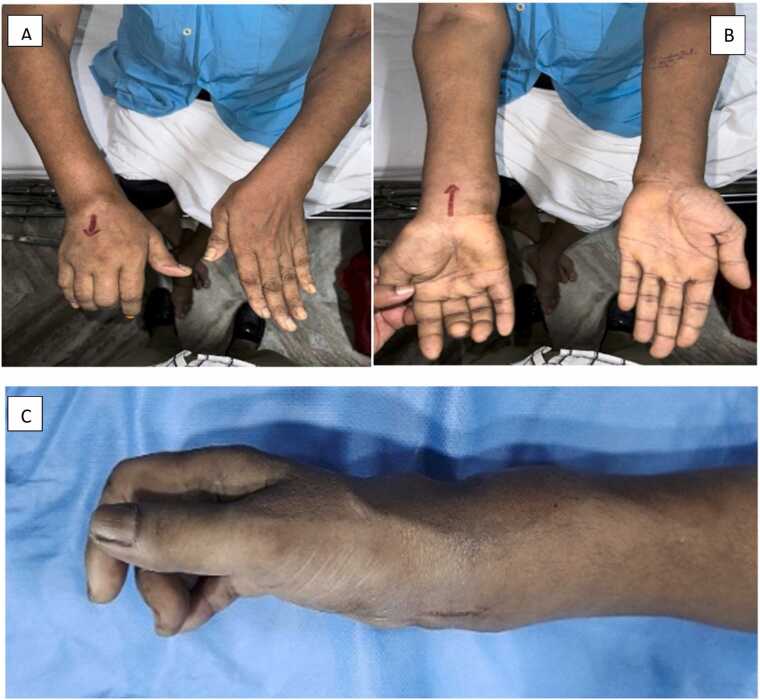
A) Right upper limb – swelling over dorsal and palmar wrist with flexion contracture of middle and ring finger, B) Right upper limb – swelling over dorsal and palmar wrist with flexion contracture of middle and ring finger, C) Lateral view of right wrist with multiple swellings leading to deformity.

Physical examination revealed normal vital signs and system examination. Local examination revealed a prominent dorsal wrist swelling measuring 5 × 6 cm, with a smaller swelling of 3 × 3 cm over the palmar aspect of the forearm (Fig. 1.A). Flexion contracture of the right middle and ring fingers was observed, and movement of the wrist was limited (Fig. 1.B, Fig. 1.C).

Initial blood examinations revealed normal levels of serum C-reactive protein and haemoglobin as well as total white blood cell and platelet counts. Hepatic and renal function test results were normal. Erythrocyte sedimentation rate (ESR) and blood HbA1c levels were 105 mm/hr and 10, respectively. The Mantoux test results were negative. Tests for the rheumatoid factor, anti-nuclear antibodies (ANAs), and anti-cyclic citrullinated peptide (CCP) were negative. Paired blood cultures sent were negative. Chest radiography revealed minimal bilateral infiltration. Ultrasonography of the right axilla revealed multiple lymph nodes with a preserved fatty hilum. X-ray of the right wrist revealed diffuse phalangeal osteopenia. Magnetic resonance imaging (MRI) of the right hand had been performed at an outside facility, and their written report described diffuse inflammatory changes involving the tendon sheaths of flexor pollicis longus, flexor digitorum profundus, extensor digitorum, extensor indices tendon sheaths, extensor digiti minimi and extensor carpi ulnaris tendons. Repeat MRI was not performed due to financial constraints.

Considering multiple soft tissue swellings and fever in an individual with uncontrolled diabetes with a low serum CRP level and elevated ESR, we suspected a fungal or tubercular aetiology. Other possible causes of inflammatory fever with an elevated ESR in a middle-aged woman included rheumatoid arthritis and connective tissue disorders; however, the patient lacked classical symptoms, and test results for these conditions were negative. An abdominal ultrasonography was performed to exclude organomegaly. Sexually transmitted infections such as HIV, hepatitis B, and syphilis were negative. We also considered the possibility of an underlying malignancy with cutaneous deposits.

**Fig. 2:**
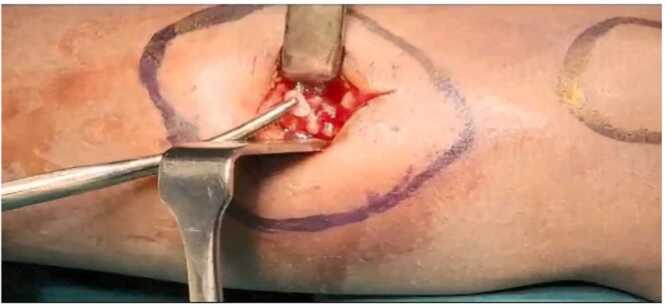
Intra operative image showing rice bodies.

Orthopaedics opinion was obtained, and patient was taken up for exploration and intraoperative picture showing rice bodies of the tendon sheath (Fig. 2).

**Fig. 3:**
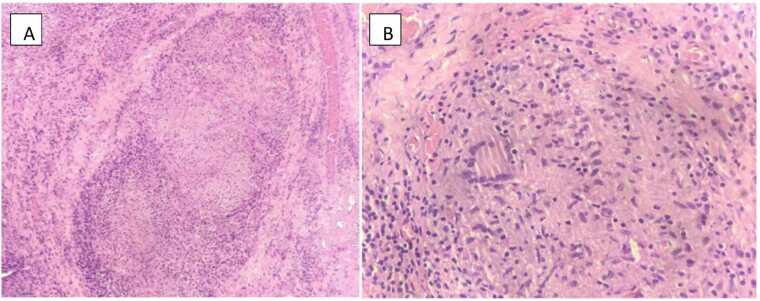
A: Histopathology image of the synovial tissue, B: Langhans giant cells on histopathology.

Tissue samples sent for acid-fast bacilli (AFB) smear, AFB culture and nucleic acid amplification test (NAAT- Gene Xpert Ultra) for M.tuberculosis complex were positive. In view of chest Xray showing infiltrates, sputum was sent for smear AFB which was negative while culture AFB and NAAT were positive for M.tuberculosis complex. Histopathology of tissue biopsy showed synovial tissue infiltrated by chronic inflammatory cells with multiple well-formed granulomas composed of epithelioid histiocytes with Langhans type multinucleate giant cells surrounded by lymphocytes and plasma cells (Figs. 3A, 3B).

The patient was initiated on anti-tubercular therapy (ATT): isoniazid 300 mg, rifampicin 600 mg, pyrazinamide 1500 mg and ethambutol 900 mg every day for 2 months. This was followed by a three- drug phase: isoniazid 300 mg, rifampicin 600 mg, and ethambutol 900 mg every day for 10 months. Physiotherapy and supportive care were also continued.

After 4 weeks of ATT, repeat sputum smear was negative for AFB. On follow-up after 2 months of ATT, the patient was symptomatically better and continued physiotherapy. She was able to move her right wrist freely and perform day-to-day activities, such as holding a pen and buttoning her shirt, which the patient previously found to be painful. She completed 12 months of ATT and is currently off medications and doing well.

## Discussion

Tuberculosis, otherwise known as “white death” or “consumption”, caused by *Mycobacterium tuberculosis,* is a major cause of mortality and morbidity among the developing countries. Globally, an estimated 10 million people develop tuberculosis, and over a million deaths occur annually [1]. Pulmonary TB accounts for nearly 85 % of reported cases, while EPTB represents a significant clinical burden, particularly in immunocompromised populations [1], [2]. TB that affects any part of the body other than the lungs is known as extrapulmonary TB, with lymph node involvement being the most common [1], [2].

The coexistence of pulmonary tuberculosis and extrapulmonary Tenosynovial involvement in our patient satisfies the definition of disseminated tuberculosis involving two non-contiguous sites via lymphohematogenous spread [1]. Pulmonary involvement was microbiologically confirmed by sputum culture and nucleic acid amplification testing despite an initial negative smear.

TB presenting primarily as tenosynovitis is a rarity [4].Tenosynovitis is characterised by chronic granulomatous inflammation caused by *M.tuberculosis*. Susceptible individuals include patients with impaired cell-mediated immunity, those on immunosuppressive drugs, those with a history of malnourishment or exposure to TB, and those with traumatic injury [2].Tuberculous tenosynovitis typically spreads via direct inoculation, local spread from the adjacent bones, or haematogenous spread from the lungs [4].

Recent case series and contemporary review have confirmed that tuberculous tenosynovitis constitute a very small proportion of extrapulmonary tuberculosis cases and is frequently associated with delayed diagnosis due to its indolent onset and non-specific clinical features [6], [7].

The formation of rice bodies or melon bodies is an important pathological characteristic of tuberculous tenosynovitis [8]. These are small, white, rice-like particles observed in the synovial fluid, comprising fibrinous material, and are considered hallmarks of chronic synovial inflammation [9]. The immune response against mycobacteria causes the aggregation of macrophages, T lymphocytes, and multinucleated giant cells, which results in the formation of granulomas. Granulomatous inflammation leads to the production of fibrinous material over time, which eventually coalesce into rice bodies. They are thought to arise from

micro infarction after intra articular synovial inflammation and ischemia after which they shed into the articular or bursal fluid [9]. The infection appears to begin in the synovium and gradually progresses from serous exudate in tendon sheaths to the sero-fibrinous stage, thereby causing abscess formation, which leads to tendon rupture and even bone involvement [9]. Rice bodies are also observed in inflammatory conditions such as rheumatoid arthritis, seronegative arthritis, and arthritis of other infectious aetiologies [10].

Common clinical presentations include swelling along the affected tendon sheaths, pain, and tenderness with reduced range of mobility of the joint due to swelling and pain. Weight loss and low- grade fever may also occur. The most affected sites are the hands, followed by the ankles and feet [11].

Tuberculosis cases with non-specific clinical presentation are challenging to diagnose. Imaging modalities such as ultrasonography and MRI can help identify tenosynovial thickening and the presence of rice bodies [12], [13].

The Mantoux (tuberculin skin) test has limited sensitivity in extrapulmonary tuberculosis and in immunocompromised states such as diabetes mellitus, resulting in false negative reactions [2], [6]. This was evident in our patient, whose Mantoux test was negative despite microbiologically confirmed tuberculosis. Interferon – gamma release assays (IGRAs) generally offer higher specificity for *mycobacterium tuberculosis* infection, as they are unaffected by prior Bacillus Calmette-Guerin (BCG) vaccination or exposure to most nontuberculous mycobacteria and may serve as a useful adjunct in such settings [7].

Recent imaging-based reviews emphasise MRI as the preferred modality for diseases staging, assessment of disease extent, and surgical planning, particularly in differentiating tuberculous tenosynovitis from inflammatory and neoplastic conditions [14]. In our case, MRI had been performed at an outside facility and the written report was reviewed; however, the imaging films were not available for reassessment or publication. Repeat MRI was not performed due to financial constraints, a limitation frequently encountered in clinical practice in resource – limited settings. Preoperative evaluation was therefore based on detailed clinical examination and intraoperative assessment. The diagnosis and extent of disease were ultimately confirmed by surgical exploration and histopathological examination.

Tendon sheath tuberculosis consists of three stages – serous exudation due to sheath thickening (stage-1), proliferative phase of granulomatous tissues forming rice bodies (stage-2) and necrosis with abscess formation (stage- 3) [3], [13]. Our patient presented in the second stage. Our patient also had axillary lymphadenopathy suggestive of tubercular lymphadenitis.

Diagnosis is usually confirmed by the identification of M.tuberculosis in the synovial fluid, AFB staining of the biopsied tissue, NAAT for M.tuberculosis DNA. Histopathological evidence of granulomatous inflammation with caseating necrosis is a characteristic of tuberculosis [13], [15].

The mainstay treatment for tuberculous tenosynovitis involves a combination of surgical intervention and first-line anti-TB drugs. ATT comprises an intensive two-month phase of treatment with isoniazid, rifampicin, pyrazinamide, and ethambutol, followed by a 8–10 month phase of treatment with isoniazid, rifampicin, and ethambutol [14], [15]. Recent studies have demonstrated that prolonged antitubercular therapy combined with timely surgical debridement is associated with favourable functional outcomes and low recurrence rates, particularly when treatment is initiated before tendon rupture or extensive necrosis occurs [16]. Surgical intervention is beneficial in cases with extensive involvement of the synovium, abscess formation, or failure of medical therapy and accelerates improvement. Rice bodies regardless of any cause should be removed as presence of fibrin, which is a known irritant of synovial tissue, will act as a stimulant for continuous synovial inflammation [17]. In our case, we reviewed the previous literature on treatment of tb tenosynovitis of the wrist and hand (Table 1). The general prognosis of tuberculous tenosynovitis is good if it is diagnosed early, and treatment is initiated. Early intervention is crucial to prevent irreversible damage [16].

**Table 1:** Summary of previously published studies on tuberculous tenosynovitis of the hand and wrist.

**Author**	**Year of publishing**	**Location**	**Article type**	**Patient risk factors**	**Presence of rice bodies**	**Treatment**	**Outcome**
Oshima M et al. [17].	2004	Japan	Case report	Systemic lupus erythematosus; long term steroid therapy	Not reported	Debridement + ATT	Good outcome
Marques VB et al. [18].	2010	Brazil	Case report	Not reported	Not reported	Surgery + ATT	Good outcome
Woon CY-L et al. [19].	2011	Singapore	Case series (6 cases)	Immunosuppression in 4/6 patients	Present	Debulking tenosynovectomy + ATT	No recurrence
Chavan S et al. [20].	2012	India	Case report	Not reported	Present	Excision + ATT	Good outcome
Probst FA et al. [21].	2012	Germany	Case report	Not reported	Not reported	Tenosynovectomy + ATT	Good outcome
Sbai MA et al. [22].	2015	Tunisia	Case report	Not reported	Not reported	Synovectomy + ATT	Good outcome
Bayram S et al. [23].	2016	Turkey	Case report	Not reported	Present	Extensive debridement +ATT	Good outcome
Cohen-Tanugi S et al. [24].	2018	Usa	Case report	Not reported	present	Synovectomy + ATT	Good outcome
Reddy GP et al. [25].	2018	India	Case report	Not reported	Present	Synovectomy + ATT	Good outcome
Hogan JI et al. [26].	2019	Usa	Case report	Not reported	Not reported	ATT	Not available
Takahashi M et al. [27].	2019	Japan	Case report	Not reported	Not reported	Tenosynovectomy + ATT	Good outcome
Suwannaphisit S et al. [7].	2020	Thailand	Case report	Not reported	Not reported	ATT + Surgery	Good outcome
Korkmaz MC et al. [28]	2021	Turkey	Case report	Not reported	Not reported	Synovectomy +ATT	Good outcome
Sharma A et al. [29].	2021	India	Case report	Not reported	Not reported	Synovectomy +ATT	Good outcome
Giri SK et al. [30].	2023	India	Case report	Not reported	Present	Synovectomy +ATT	Good outcome
D’Souza A et al. [31].	2022	India	Case report	Not reported	Not reporter	Debridement + ATT	Good outcome
Ngoc CT et al. [32].	2023	Vietnam	Prospective study (25 cases)	Not individually specified	Present	ATT (9–12 months)	Good outcome
Abdulrahman BB et al. [33].	2023	Iraq	Case report	Not reported	Not reporter	Surgery + ATT	improved
Singh M et al. [34].	2023	India	Review article	Not reported	Not reporter	Surgery + ATT	Good outcome
Chahal JS et al. [35].	2024	India	Case report	Not reported	Not reporter	ATT	Good outcome
Gomez D et al. [36].	2024	Sri Lanka	Case report	Systemic lupus erythematosus	present	Synovectomy + ATT	Good outcome
Basnayake O et al. [37].	2024	Sri Lanka	Case report	Rheumatoid arthritis	Not reported	Surgery + ATT	Good outcome
Kataria S et al. [38].	2025	India	Case report	Not reported	Not reported	ATT	Good outcome
El Aissaoui T et al. [39].	2025	Morocco	Case report	Not reported	Not reported	ATT	Good outcome
Siham O et al. [40].	2025	Morocco	Case report	Not reported	present	Surgery + ATT	Improved

Not reported indicated information not specified in original publication.

## Conclusion

Tuberculous Tenosynovitis, even though uncommon can still occur in TB endemic areas. Its subtle onset with ambiguous clinical picture makes the diagnosis difficult. Microbiology, imaging, and laboratory analysis is useful in making the diagnosis. Mainstay treatment consists of anti-tubercular drugs, however in more complex and advanced cases surgery may be required. This case highlights tuberculous tenosynovitis as a potential sentinel manifestation of disseminated tuberculosis, particularly in patients with uncontrolled diabetes.

## CRediT authorship contribution statement

**Merlin Moni:** Supervision, Investigation, Conceptualization. **Balu Chandrababu:** Supervision, Investigation. **Nivedita Suresh:** Writing – review & editing, Investigation. **Anna Kurian:** Writing – review & editing, Resources, Investigation. **Anil Kumar:** Writing – review & editing, Supervision, Investigation. **Satyapalan Dipu T:** Writing – review & editing, Supervision, Conceptualization. **Kulirankal Kiran G:** Writing – review & editing, Writing – original draft, Supervision, Investigation, Conceptualization. **Thomson Eunice:** Writing – original draft, Resources, Data curation.

## Author Agreement Statement

We, the undersigned authors, hereby declare that the manuscript entitled:

“Tuberculous tenosynovitis as the initial presentation of disseminated tuberculosis in a patient with uncontrolled diabetes: a case report and review of literature”

is original, has not been published previously, and is not under consideration for publication elsewhere.

All authors have made substantial contributions to the conception and design of the work, acquisition, analysis, or interpretation of data, drafting or critically revising the manuscript, and have approved the final version to be submitted.

We confirm that:

• The manuscript is original work.
• All authors have approved the submitted version.
• The order of authors has been agreed upon by all contributors.
• There are no conflicts of interest related to this work.
• If accepted, the article will not be published elsewhere in the same form, in English or any other language, without written consent of the copyright holder.

The corresponding author is responsible for communicating with the journal during the submission, review, and publication process.

## Declaration of Competing Interest

The authors declare that they have no known competing financial interests or personal relationships that could have appeared to influence the work reported in this paper.
